# Comparison of the Physical and Sensory Properties of Hybrid Citrus Fruit Jaffa^®^ Sweetie in Relation to the Parent Fruits

**DOI:** 10.3390/molecules25122748

**Published:** 2020-06-13

**Authors:** Martyna Lubinska-Szczygeł, Żaneta Polkowska, Tomasz Dymerski, Shela Gorinstein

**Affiliations:** 1 Department of Analytical Chemistry, Faculty of Chemistry, Gdansk University of Technology, 80-233 Gdansk, Poland; tomasz.dymerski@pg.edu.pl; 2 Institute for Drug Research, School of Pharmacy, Faculty of Medicine, The Hebrew University of Jerusalem, Jerusalem 9112001, Israel; shela.gorin@mail.huji.ac.il

**Keywords:** flavoromics, fruit hybridization, gas chromatography, sensory analysis, terpenes

## Abstract

In the presented study, an overall Jaffa sweetie evaluation was made to find a correlation between *Citrus grandis* Osbeck *× Citrus paradisi* Macf. and its parent fruits’ (*Citrus grandis* Osbeck, *Citrus paradisi* Macf.) properties. Based on the sensory analysis, it was found that the taste and aroma of the new hybrid fruit are close to pummelo. By the use of chromatographic analysis, the selected monoterpenes present in the fruits were quantified. α-terpineol was typed as the main monoterpene compound in the headspace of sweetie and grapefruit, with the concentrations: 20.96 and 87.9 μg/g, respectively. In turn, γ-terpinene was chosen as the most important monoterpene determining the flavor of sweetie fruit. Based on two-dimensional gas chromatography (GC × GC-TOF-MS) and principal component analysis (PCA) of the data, several volatile compounds were associated with analyzed fruits’ aroma. Jaffa Sweetie is the hybrid fruit with sensory properties similar to pummelo with a higher content of monoterpenes, which improves its health benefits compared to the parent fruit. The research presents an instrumental method for assessing the aroma properties of the fruit as a reference method for sensory analysis, commonly used in the industry.

## 1. Introduction

Fruits are important elements of the human diet. According to the latest recommendations of dietitians, several portions of fruit should be consumed every day. Due to the consumers’ willingness for the consumption of fruits with the greatest health benefits, scientists and fruit farmers are still looking for new plant varieties that meet the expectations related to the content of vitamins or other substances with health-promoting effects. From the point of view of fruit farmers and producers, the new fruits should represent some specific functional properties, such as higher yields, greater resistance to climatic factors, or lack of seeds [1]. One of the popular solutions, used for many years and gaining more and more popularity, is the production of hybrid plants. Cross-breeding, also called fruit hybridization, is the botanical mating of two different plant species or varieties to create the hybrids that have all the best qualities of parent plants and none of their defects [2]. Hybrid plants can be created by cross-breeding individual varieties or species. Hybridization within one species can lead to a phenomenon known as heterosis. Heterotic individuals are characterized by higher fertility, better lifespan, and higher fruitfulness. The newly-created fruits, despite functional properties and health properties, also have new organoleptic attributes [3]. 

One of the popular hybrid fruit in recent years is sweetie (*Citrus grandis* Osbeck *× Citrus paradisi* Macf.), also called oroblanco, a hybrid between the giant orange, called pummelo (*Citrus grandis* Osbeck) and grapefruit (*Citrus paradisi* Macf.). The fruit was patented in 1981 by scientists at the University of California at Riverside [4]. This hybrid was created to improve the taste qualities of grapefruit and functional properties of pummelo while maintaining the nutritional properties. According to the reports, in 2019 sweeties market outlook in China was very promising in the long run [5]. Gorinstein et al. provided a complementary characteristic of bioactive properties of sweetie, by the determination of total phenol content and the antioxidant activity which was higher in oroblancos than in grapefruits [6]. 

The taste and the aroma of the fruit are very important factors determining the consumption of fruit by the consumers. It is also essential to determine the content of chemical compounds with pro-health effects, such as terpenes, polyphenols, anthocyanins, and flavonoids [7,8]. Determining the physical characteristics of the fruit is also useful when planning technological or logistic processes. In addition, based on the determination of the color of the fruit’s peel, the preliminary evaluation of fruit’s quality or freshness is possible. As there are four main characteristics that impart distinctive quality to the fruits: (1) color and appearance, (2) flavor (taste and aroma), (3) texture, and (4) nutritional value [9], the goal of the research was to show the new approaches in the evaluation of visual and flavor properties of Jaffa sweetie and comparing with its parent fruits to provide a full characteristic of the fruit. The second objective was the assessment of the effectiveness of the hybridization process of the oroblanco fruit from the consumers’ point of view. For this purpose, the basic physical parameters of hybrid fruits were evaluated and the sensory analysis was carried out. The comparison of monoterpenes content was provided for sweetie, grapefruit, and pummelo. Determination of this group of compounds is important not only due to their sensory attributes but also because of their bioactive properties which complements the research previously conducted in terms of nutritional values. All chromatographic analyses were conducted using two-dimensional gas chromatography with mass spectrometry (GC × GC-MS) which has been widely used for the analysis of a variety of complex food samples. According to the best of our knowledge, no work has been performed so far to evaluate the monoterpenes content in Jaffa sweetie fruit and its parents’ fruits using GC × GC-MS. The provided analysis of sweetie supplements the research on the bioactive properties of sweetie [6] in the full characteristics of this fruit. The results ensure a background for further industrial purposes. 

## 2. Results and Discussion

Quality and consumers’ acceptance of fruits depends on several attributes, such as color, appearance, flavor, texture, and nutritional properties. First of all, the investigation strategy involves determining its nutritional properties, as well as the chemical compounds responsible for flavor and aroma, and defining the degree of acceptance of the fruit by consumers in organoleptic tests. Moreover, by comparing the attributes of hybrid fruits with their parent fruits, the effectiveness of the crossbreeding process can be evaluated. 

### 2.1. Overall Visual Fruit Evaluation

The first stage of the analysis was the overall visual assessment of the fruits, as well as the measurement of the fruit’s weight and the outer diameter of the fruit at the widest point. The physical properties of the fruit are determined mainly as the quality of the fruit. The knowledge of some important physical properties is essential for the design of the storage structures, processing equipment, and processes [10]. The individual physical characteristics of the sweetie and its parent fruits are presented in Table 1. 

**Figure 1:**
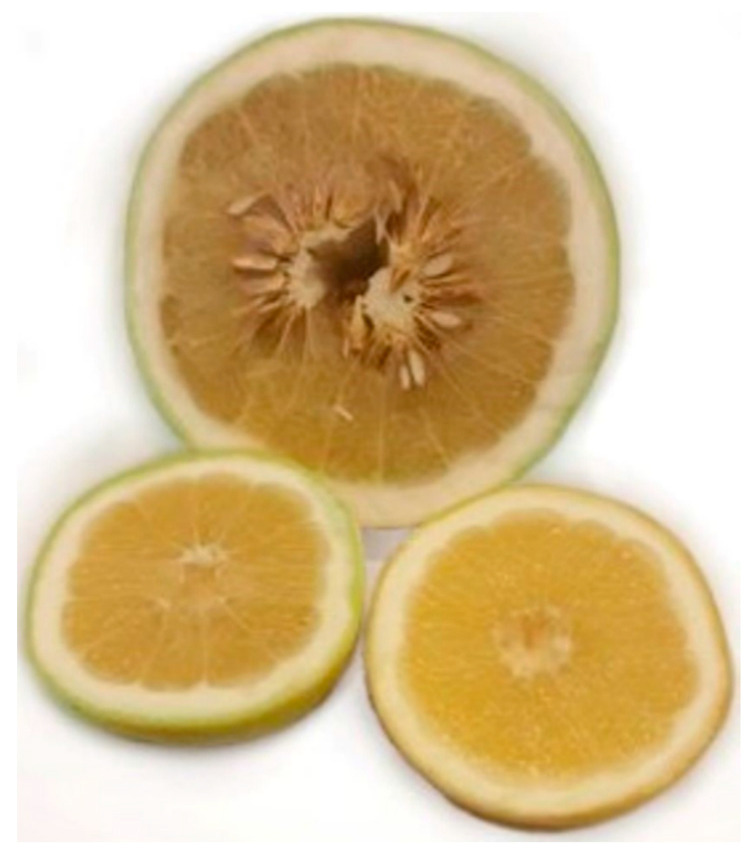
Cross-section of analyzed fruits.

The cross-section of analyzed fruits is shown in Figure 1. Considering the visual characteristics of the sweetie hybrid, it can be stated that all parameters including weight, shape, and external diameter of the fruit were inherited from the grapefruit. However, the color of peel and flesh, as well as the thickness of peel, are a combination of the characteristics of both crossbreed fruits. In this respect, sweetie appears to be a beneficial hybrid, due to the elimination of undesirable fruit properties such as peel thickness. Not without significance is the absence of seeds in the hybrid sweetie fruit, which is an advancement in science and technology especially biotechnology [11].

### 2.2. Sensory Analysis

**Figure 2:**
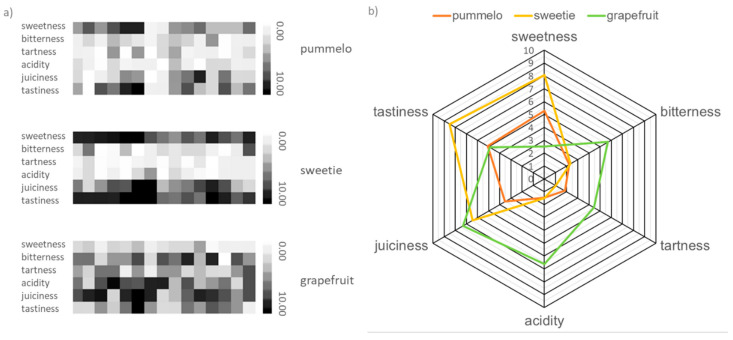
Results of sensory analysis of pummelo, sweetie, and grapefruit: (**a**) heat map of panelists’ choices and (**b**) radar plot of average values of main sensory properties.

Sensory analysis is very popular in the fruits’ analysis [12]. Previous sensory analysis of pummelo juice indicated that the most aroma-active compounds contributed to the sensory perception of acidic, fresh, and peely notes while the non-volatile components were correlated with the sour and sweet tastes [13]. In turn, the sensory analysis of grapefruit juice made it possible to find a correlation between its taste and the content of selected chemical compounds. The 1-p-menthene-8-thiol is responsible for the taste of the grapefruit-like flavor and octanal for the citrus-like flavor [14]. The results of the sensory analysis are presented in Figure 2. Members of the panel choose oroblanco hybrid as the tastiest of analyzed fruits. Panelists almost unanimously stated that it is also the sweetest fruit. Therefore, it can be concluded that this property determines the tastiness of the fruit. It is related to the total amount of sugars, which in the case of oroblanco fruit is almost twice larger than in white grapefruit (57.63 ± 2.78 mg/g FW, 108 ± 1.77 mg/g FW, respectively) [15]. The total sugars content in pummelo is 200 mg/g FW [16], so the hybridization process, in addition to improving the taste properties, resulted in a reduction of the sugar content in oroblanco fruit compared to parent fruit, pummelo. Citrus fruits are widely recommended for diabetics, so a lower sugar content and better taste of sweetie comparing to pummelo, makes the hybrid fruit even more attractive in the diabetic diet. As the second, in case of sweetness, the pummelo fruit was chosen. The bitterness (2.2 and 2.3 points on a 10-point scale) and tartness (1.9 and 1.0 points) of oroblanco are similar to pummelo. The sweetie hybrid fruit is as sour as pummelo, according to the respondents’ assessment. However, in the case of grapefruit, it was found that it is the most bitter and acidic of the fruits under evaluation. These are undesirable flavor properties in the fruit, therefore it can be concluded that this is the least tasty fruit. This is confirmed by the overall taste rating. Over half of the respondents chose this fruit as the least tasty. Grapefruit is the juiciest of the three analyzed citruses. Oroblanco got a slightly lower rating, in this category. It can be concluded that this property was originating from a grapefruit. Based on the research, it can be stated that the sweetie fruit is more similar in taste to pummelo. The new hybrid also managed to get rid of the bitter taste of grapefruit, which is an advantage when choosen by consumers. 

### 2.3. Chromatographic Analysis

**Figure 3:**
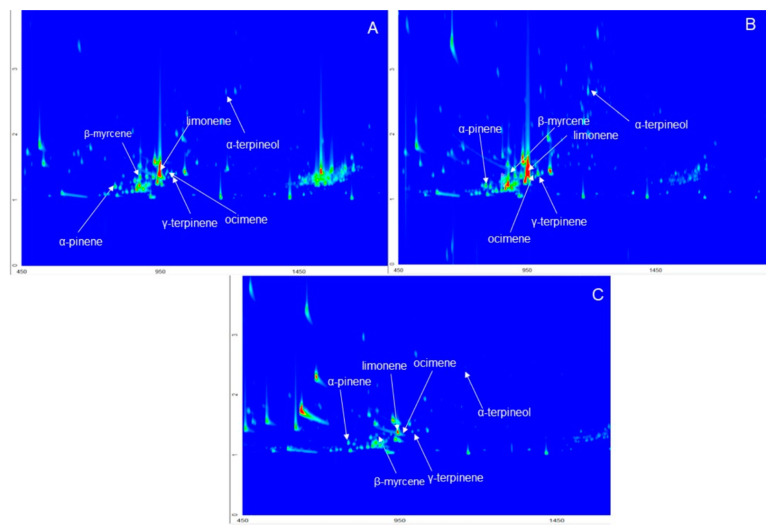
Two-dimensional gas chromatography (GC × GC) contour plots in total ion current (TIC) mode of (**A**) grapefruit, (**B**) sweetie, and (**C**) pummelo samples.

Despite the tart and bitter taste, grapefruits are still very popular and more eagerly consumed then other fruit tested. This can be associated with the lower availability of sweetie compared to grapefruits, but above all, from the general opinion about the health-promoting properties of grapefruit. Several studies have been carried out on the analysis of chemical compounds with health effects in grapefruit samples [6,14,15], pummelo [17,18,19], and sweetie [6,15,20,21]. Many of these compounds, including terpenes, flavonoids, and polyphenols can be determined using gas chromatography. For determination of compounds from the terpenes group in a complex matrix, which is food, it is reasonable to use the technique of multidimensional gas chromatography [22]. The peak capacity in GC × GC is much higher comparing the one dimensional GC, which results in a significantly improved separation of individual analytes, and their separation from interfering matrix compounds. There is a lack of scientific reports about utilizing GC × GC for volatile organic compounds (VOCs) determination of pummelo, white grapefruit and sweetie samples. Total ion chromatogram contour plots of the citrus fruits VOCs using GC × GC analysis are presented in Figure 3.

#### 2.3.1. Qualitative Analysis

Based on the provided analysis, it was possible to detect about 600 chemical compounds in samples of pummelo, grapefruit and sweetie. The main chemical compounds identified in the samples of sweetie, grapefruit, and pummelo are presented in Table 2. 

**Figure 4:**
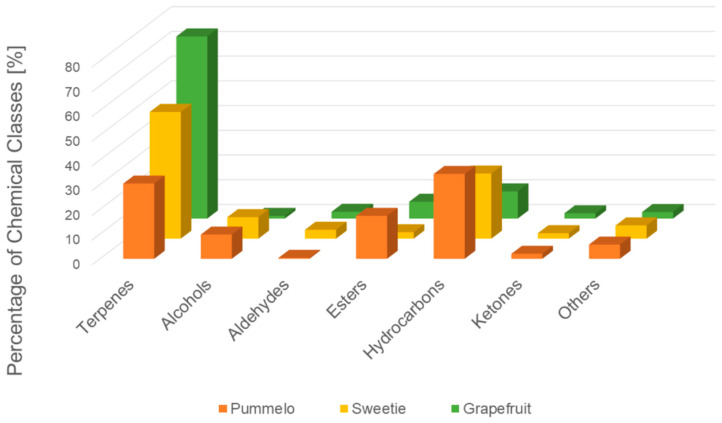
Distribution of volatiles by chemical classes.

The compounds were divided into seven chemical classes (Figure 4). Terpenes are the dominant class in the fruits’ headspace. Terpenes are secondary metabolites of many plants, produced to meet specific biological functions, such as hormone biosynthesis, but also protects against UV radiation and photooxidative stress, including pest and toxin repellents, growth regulators, pollinator attractors, photosynthetic dyes, and electron acceptor. They are the main ingredients of citrus essential oils accumulated in flavedo [23]. Citrus fruits are the main source of terpenes, especially limonene, in the human diet [24].

Human thresholds of the selected terpenes are low and, thus, they have a big influence on creating citrus aroma even though they do not constitute the largest content in citruses. Therefore, highly sensitive and selective methods for the quantification of these compounds are needed [25]. The high content of terpenes in the citrus peels can determine the bitter taste of citrus [26]. The groups of esters, alcohols, aldehydes, and ketones were significant chemical classes, regarding the amount of identified substances. These chemical substances are characterized by a specific, often intense odors. Their presence in the headspace and synergistic interactions influence the intense aroma of fruits. Among the identified chemical compounds, limonene, citronellene, and γ-terpinene are characterized by a pleasant citrus aroma [27]. The high content of terpenes in sweetie fruit makes it a valuable component of the human diet. This is in agreement with the previous results [6]. 

#### 2.3.2. Quantitative Analysis

Quantitative analysis is an essential step during the analysis of the influence of each volatile on the aroma of the sample. In the case of food samples, terpenes quantitation is also important for the understanding of the pro-health properties. For the quantitative analysis, the class of terpenes was chosen because of its greatest percentage distribution in the analyzed samples. The monoterpenes with the largest peak area were selected. The results of the quantitation of selected monoterpenes are presented in Table 3.

**Figure 5:**
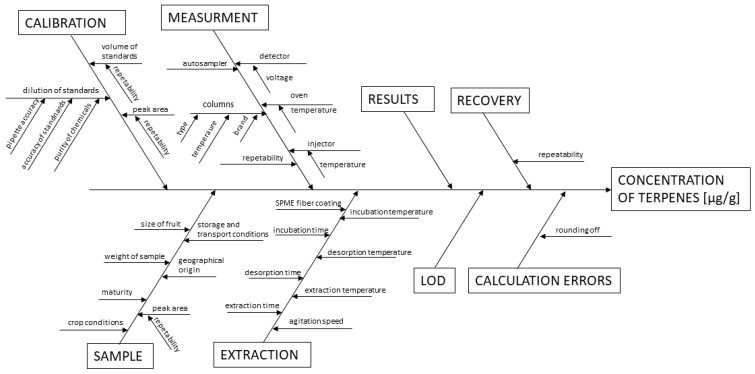
Ishikawa diagram presenting the influence of parameters on the analytical process for the determination of monoterpenes in citrus fruit samples.

Based on the quantitative determination, the aroma properties of the fruits can be explained. As the terpenes are the main group of chemical compounds in the three tested fruits, according to quantitative analysis, it can be stated that pummelo is the least aromatic of mentioned fruits. Despite the use of the two-dimensional technique, in the pummelo fruits’ flesh, it was possible to quantitatively determine only one volatile, namely limonene. The reason for this fact can be explained by the low interchange of chemicals between flesh and volatile fraction. Until now, the use of chromatographic techniques allows to determine the content of terpenes only in pummelo juices or extracts [13,28]. In contrast, in the sweetie and grapefruit volatile fraction, six terpenes were determined. In both cases, α-terpineol was the compound with the highest content. Its content was more than four-times higher in grapefruit samples. The earthy odor description of α-terpineol can be one of the reasons for the bitter flavor of grapefruit fruit. Limonene was identified and determined in all fruits, which is consistent with literature reports of Rodríguez et al. in which this volatile is described as a characteristic for citrus [29]. The highest content of limonene was determined in the grapefruit headspace, namely 15.79 ± 0.30 μg/g. The obtained results are correlated with the report of Zhang H et al. [28]. Twelve terpenes in oroblanco and three in pummelo juice samples were determined. However, it was not possible to identify α-terpineol in oroblanco samples. Nevertheless, α-terpineol may be formed in citrus fruit from limonene during biochemical processes [30]. Hence, many factors, such as growing and storage conditions, can affect this difference. In turn, Buettner et al. determined five terpenes in the samples of the yellow grapefruit juice, and the concentration of limonene was 2308 μg/kg [14]. The sources of uncertainty associated with the determination of terpenes in citrus fruit samples are presented graphically using the Ishikawa diagram (Figure 5). On the basis of the content of individual monoterpenes in the volatile fraction of the sweetie and its parent fruits flesh, it can be stated that the hybrid fruit is more aromatic than pummelo. 

Based on the quantitative analysis, the odor parameters of the selected compounds were calculated (Table 4). Almost all of the determined volatile components presented odor activity values (OAVs) greater than 1, which means that they are odor active compounds (OAC) and have a greater potential to influence samples’ aroma [31]. Ocimene (OAV—47.1 ± 2.9), limonene (OAV—26.49 ± 029), and β-myrcene (41 ± 1.4) with pleasant fruity and citrus odors are the key aroma compounds of sweetie. In the case of grapefruit, OAV values are also high for α-pinene and α-terpineol, 15.01 ± 0.08 and 17.58 ± 0.40, respectively. These volatiles with herbal and terpenic odor contribute to the aroma of grapefruit. 

### 2.4. Multivariate Analysis

**Figure 6:**
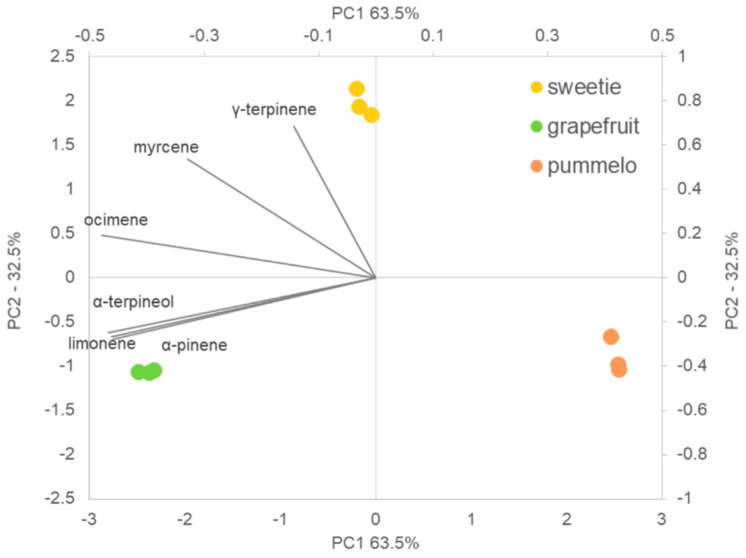
Principal component analysis (PCA) biplots of the assignments of sweetie, grapefruit and pummelo fruits in respect of individual volatile organic compounds (VOCs).

The results of the principal component analysis (PCA) that was made to distinguish between citrus fruits based on the main monoterpenes content fruits are shown in Figure 6. In the case of the dataset obtained during the analysis of citrus fruits, the first two components explained 96% of the total variance (axis 1 (63.5%) and axis 2 (32.5%)). It can be observed, that the total separation of citrus samples along the two first main components was obtained. The PCA biplot grouped samples in a distinct cluster, showing that their properties are different. However, based on the analysis and the distance between data points in multidimensional space it can be stated that sweetie is more similar to grapefruit in the context of the content of monoterpenes. Moreover, PCA-biplot allows correlating between the selected monoterpenes and the group of citrus fruits. Γ-terpinene was positively correlated with the samples of sweetie, while α-terpineol, α-pinene, and limonene were positively correlated with grapefruit. Herbal and terpenic flavor description of these compounds may explain the bitterness of grapefruit samples, which is in agreement with sensory analysis. 

## 3. Materials and Methods 

### 3.1. Sensory Analysis

Sensory analysis was carried out by the fifteen-member panel with the use of profiling method [33]. The fleshes of grapefruit, sweetie and pummelo were the subjects of the test. The evaluation of the samples was performed with the use of six descriptors which are the most common in the case of fruits analysis: sweetness, bitterness, tartness, acidity, juiciness, and tastiness. Before the test, fruits were washes with diluted water and peeled. The flesh was manually separated from the feel and from membranes which bitter taste could falsify the results. Fruit samples were coded by the person carrying out the analysis. Panelists graded the perceived hedonic quality on a 10-mm-long axis, with 10 and 0 denoting the most and least desirable qualities, respectively. The final assessment was based on the average of values set by the panelists. Section lengths were measured by caliper.

### 3.2. Sample Preparation for Chromatographic Analysis

**Figure 7:**
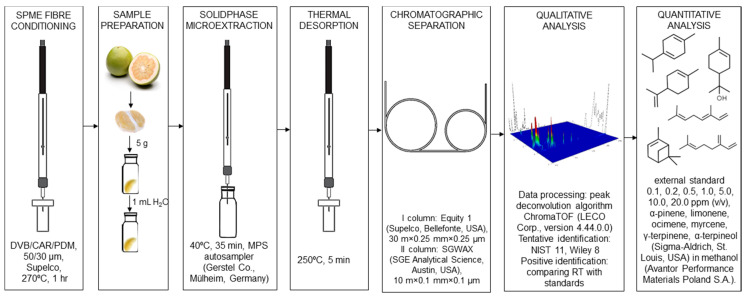
The process of sample preparation for analysis.

The fruits for analysis were purchased at local distribution points in the Pomeranian Voivodship. From the information provided by the supplier, it appeared that the fruit was harvested in a similar degree of maturity and that the time since harvest was the same. Samples were analyzed immediately after purchasing. Solid phase microextraction was used for the isolation and enrichment of analytes. Analysis of each fruit was conducted in triplication, each time using the new fruit from each variety from a different supplier. The scheme of sample preparation is shown in Figure 7.

### 3.3. GC × GC-TOF-MS Analysis

Samples of citrus fruits were analyzed using two-dimensional gas chromatography. The utilized apparatus consists of Agilent 7980A chromatograph (Agilent Technologies, Palo Alto, CA, USA), equipped with a dual-stage cryogenic modulator which was coupled with a time-of-flight mass spectrometer (LECO Corp., St. Joseph, MI, USA). The parameters and conditions of chromatographic analysis are shown in Table 5. 

### 3.4. Data Processing and Statistical Analysis

Data processing was performed using chromatographic peak deconvolution algorithm implemented in the software ChromaTOF (LECO Corp., version 4.44.0.0, St. Joseph, MI, USA). Tentative identification of analytes was made by comparing experimental spectra with the spectra included in National Institute of Standards and Technology (NIST) 11 and Wiley libraries. Microsoft^®^ Excel^®^ spreadsheet was used for data entry and calculations. LOQ and LOD were calculated at the materiality level α = 0.05. The LOD values were calculated based on the residual standard errors of the calibration curve (SE) and slope of the curve (a): LOD = 3.3 SD/a. LOQs values were calculated as three LOD. Odor activity values (OAV) were calculated as a ratio of the mean concentration of selected compounds and their odor threshold values taken from literature. Chromatographic peak areas for 6 selected chemical compounds were used as input data for Unsupervised Principal Component Analysis (PCA). Statistical analysis was performed using Orange v.3.8.0 software ((Bioinformatics Lab, University of Ljubljana, Slovenia).

## 4. Conclusions

In the present work, GC × GC-TOF-MS and sensory analysis were used to evaluate the flavor properties of Jaffa Sweetie and its parents’ fruits. Moreover, the physical characteristics were assessed. The main attributes of *Citrus grandis* Osbeck *× Citrus paradisi* Macf., *Citrus paradisi* Macf., and *Citrus grandis* Osbeck were compared. It was shown that the visual properties of sweetie were originating from the grapefruit. Based on the obtained results of the sensory panel, it can be concluded that the taste and aroma of sweetie are most desirable by consumers and closer to pummelo. Considering the concentration of individual monoterpenes, it was proved that sweetie is a hybrid in which the health-promoting properties of the grapefruit were preserved. The content of individual monoterpenes is higher than in pummelo. α-Terpineol with the concentration of 20.96 ± 0.70 μg/g is the most abundant monoterpene in the volatile fraction of sweetie, notwithstanding ocimene with pleasant fruity odor is the monoterpene with the greatest influence on the sweetie aroma because of the high value of odor activity value. In addition, the functional properties of the hybrid fruit were improved in contrast to pummelo. Sweetie has a thinner peel, is juicier, and contains no seeds. All these properties make the fruit a rich source of health-promoting compounds with a pleasant taste. This study has proven the purpose and effectiveness of cross-breeding of sweetie fruit. With the reports of the results of bioactive compounds obtained previously, the conducted research will provide the complex characteristics of the fruit, proving a background for application and trade of sweetie fruit. Nevertheless, due to the numerous biochemical changes that occur in the fruit over time, which may affect the change in monoterpenes’ content, further research on citrus fruit should focus on monitoring the changes in monoterpenes concentration over time. Moreover, the conducted research focused on commercially available fruits, which is a certain limitation due to the lack of complete certainty as to the degree of fruit maturity, therefore further research including fruit analysis immediately after harvest taking into account post-harvest conditions and degree of maturity should be carried out. 

## Figures and Tables

**Table 1:** Physical and visual evaluation of the citrus fruits.

Property of Fruit	Sweetie	Grapefruit	Pummelo
Diameter at the widest point [mm]	129.7 ± 5.1	145.3 ± 7.2	189 ± 11
Peel color	yellow-green	yellow	light green
Thickness of peel [mm]	17.00 ± 0.80	16.70 ± 0.40	19.5 ± 1.2
Flesh colors	grey-yellow	yellow	straw
Presence of seed	not noticed	few	many
Shape	round, slightly flattened	round, slightly flattened	round, elongated
Weight [g]	259 ± 27	283 ± 10	875 ± 86

**Table 2:** Identification of selected volatiles compounds in sweetie, pummelo and grapefruit samples using HS-GC × GC-TOFMS technique.

Chemical Compound	CAS Number	Average		ID	S	G	P	Odor Type	Flavor Type
		RT1 [s]	RT2 [s]						
	Terpenes								
p-Menthane	99-82-1	862	1.16	MS	+	+	+	pine	n.d.
p-Cymene	99-87-6	938	1.58	MS	+	+	+	terpenic	terpenic
Ocimene	6874-44-8	970	1.41	MS, RT	+	+	+	fruity	n.d.
γ-Terpinene	99-85-4	926	1.36	MS, RT	+	+	+	terpenic	terpenic
β-Myrcene	123-35-3	878	1.38	MS, RT	+	+	+	spicy	woody
Limonene	138-86-3	950	1.38	MS, RT	+	+	+	citrus	citrus
α-Pinene	80-56-8	790	1.22	MS, RT	+	+	+	herbal	woody
Citronellene	2436-90-0	806	1.20	MS	+	+	+	floral	n.d.
β-Pinene	127-91-3	858	1.30	MS, RT	+	+	+	herbal	pine
α-Terpineol	98-55-5	1186	2.67	MS, RT	+	-	+	terpenic	citrus
	Alcohols								
Hexanol	111-27-3	654	3.47	MS	+	+	+	herbal	green
Pentanol	71-41-0	486	3.66	MS	+	+	+	fermented	fusel
3-Hexenol	928-97-2	638	0.15	MS	+	+	+	green	green
2-Hexenol	2305-21-7	658	0.27	MS	+	+	+	fruity	fruity
Octanol	111-87-5	1002	2.69	MS	+	+	+	waxy	waxy
	Aldehydes								
Hexanal	66-25-1	518	1.84	MS	+	+	+	green	green
Heptanal	111-71-7	702	1.79	MS	+	+	+	green	solvent
Nonanal	124-19-6	1046	1.70	MS	+	+	+	aldehydic	aldehydic
Octanal	124-13-0	878	1.76	MS	+	+	+	aldehydic	aldehydic
	Esters								
Ethyl 2-methylbutyrate	7452-79-1	626	1.44	MS	+	+	+	fruity	fruity
Ethyl butanoate	105-54-4	534	1.56	MS	+	+	+	fruity	fruity
Ethyl hexanoate	123-66-0	882	1.50	MS	+	+	+	fruity	fruity
Ethyl isobutyrate	97-62-1	470	1.43	MS	+	+	+	fruity	ethereal
Ethyl octanoate	106-32-1	1190	1.49	MS	+	+	+	waxy	waxy
	Hydrocarbons								
2,6-Dimethyl-2,6-octadiene	2792-39-4	902	1.23	MS	+	+	+	n.d.	n.d.
Octane	111-65-9	554	1.08	MS	+	+	+	gasoline	n.d.
Nonane	111-84-2	734	1.08	MS	+	+	+	gasoline	n.d.
4-Decene	19689-18-0	766	1.10	MS	+	+	+	n.d.	n.d.
Tetradecane	629-59-4	1362	1.10	MS	+	+	+	n.d.	n.d.
	Ketones								
3-Octanone	106-68-3	854	1.68	MS	+	+	+	herbal	mushroom
6-Methyl-5-hepten-2-one	110-93-0	850	2.06	MS	+	+	+	citrus	n.d.
2-Heptanone	110-43-0	686	1.82	MS	+	+	+	cheesy	cheesy
4-Nonanone	4485-09-0	998	1.58	MS	+	−	+	n.d.	n.d.
6-Dodecanone	6064-27-3	1482	1.56	MS	+	−	+	n.d.	n.d.
	Others								
2-Pentylfuran	3777-69-3	874	1.55	MS	+	+	+	fruity	green

ID—Method of identification: MS—identification by comparison with National Institute of Standards and Technology (NIST) mass spectral libraries; RT—identification by comparison with the retention time of analytical standard compound; Odor and flavor types were taken from The Good Scents Company; S—Sweetie, P—Pummelo, and G—Grapefruit, +/- — detected/not detected.

**Table 3:** Quantitation of selected monoterpenes present in the volatile fraction of sweetie, pummelo, and grapefruit (μg/g).

Monoterpene	R^2^	S	P	G	LOQ	LOD
α-Pinene	0.999	0.8241 ± 0.0096	<LOQ	2.851 ± 0.015	0.657	0.219
Limonene	0.996	5.298 ± 0.058	2.75 ± 0.54	15.79 ± 0.30	1.416	0.472
Ocimene	0.995	1.600 ± 0.097	<LOQ	2.057 ± 0.078	1.504	0.501
β-Myrcene	0.991	4.1 ± 0.14	<LOQ	3.22 ± 0.029	2.077	0.692
γ-Terpinene	0.997	7.27 ± 0.34	<LOQ	2.566 ± 0.026	1.152	0.384
α-Terpineol	0.992	20.96 ± 0.70	<LOQ	87.9 ± 2.0	1.928	0.643

LOQ—Limit of quantitation, LOD—Limit of detection, S—Sweetie, P—Pummelo, and G—Grapefruit.

**Table 4:** Selected volatile compounds determined in sweetie, grapefruit, and pummelo with their respective odor threshold and odor active compounds (OAC).

Chemical Compound	OT [ppm]	Sweetie	Pummelo	Grapefruit
		OAV + SD [-]		
α-Pinene	0.19	4.335 ± 0.051	-	15.01 ± 0.080
Limonene	0.2	26.49 ± 0.29	13.8 ± 2.7	78.95 ± 1.5
Ocimene	0.034	47.1 ± 2.9	-	60.5 ± 2.3
β-Myrcene	0.1	41 ± 1.4	-	32.20 ± 0.29
γ-Terpinene	0.26	28.0 ± 1.3	-	9.86 ± 0.10
α-Terpineol	5	4.2 ± 0.14	-	17.58 ± 0.40

OT—odor threshold values were taken from the literature [32].

**Table 5:** Parameters and conditions of GC × GC-TOF-MS analysis.

Element	Parameter	Value
Carrier gas	Hydrogen	1 mL/min
Front inlet	Temperature	250 °C
Temperature program	Initial temperature	I. column 40 °C
		II column 45 °C
		Modulator 60 °C
	Time to maintain the initial temperature	I, II column, modulator 210 s
	Temperature rate	I, II. column, modulator 6 °C/min
	Final temperature	I column 250 °C
		II. column 255 °C
		Modulator 265 °C
	Time to maintain the set temperature	I column 300 s
		II column, modulator 350 s
Modulation	Modulation period	4 s
Modulation	Hot pulse time	0.80 s
Modulation	Cool time between stages	1.20 s
Cooling medium	Type of medium	Liquid nitrogen
Detector	Mass range	40–400 u
Detector	Voltage	1600 V
Detector	Acquisition rate	125 spectra/s
Detector	Electron Energy	−70 V
